# Management of Pemphigus in COVID-19 Pandemic Era; a Review Article

**Published:** 2020-04-18

**Authors:** Fahimeh Abdollahimajd, Mohammad Shahidi-Dadras, Reza M Robati, Sahar Dadkhahfar

**Affiliations:** 1 Skin Research Center, Shahid Beheshti University of Medical Sciences, Tehran, Iran.; 2 Department of Dermatology, Loghman Hakim Hospital, Shahid Beheshti of Medical Sciences.

**Keywords:** COVID-19, pemphigus, severe acute respiratory syndrome coronavirus 2, therapy

## Abstract

The novel coronavirus is rapidly spreading around the world. Since the public announcement of the COVID-19 outbreak, several concerns have been raised by dermatologists as well as pemphigus patients who take immunosuppressive drugs. In this paper, we review the literature about the common treatment of pemphigus with a focus on the lessons from similar epidemics to find a proper suggestion to manage pemphigus in the COVID-19 pandemic era. The effect of many of the drugs used for treatment of Pemphigus vulgaris (PV) on COVID-19 is not clear. We also do not have data on the impact of this autoimmune disease, which may involve the mucous membranes, on the acquisition or course of COVID-19. We are currently in the midst of a pandemic and evaluating the effect of COVID-19 on the population of susceptible patients suffering from auto-immune diseases like pemphigus is essential. The evidence on best ways to manage patients with underlying conditions, such as pemphigus, during the outbreak of COVID-19 is evolving and the data is updated every day.

## Introduction

The 2019 novel coronavirus (Severe Acute Respiratory Syndrome Coronavirus 2 [SARS-CoV-2]) is spreading around the world and has caused a pneumonia outbreak originating in Wuhan, China. The disease was later named coronavirus disease 2019 (COVID-19) in February 2020, by WHO (1). The epidemiological and clinical characteristics of patients, as well as risk factors for mortality and clinical course of illness have been illustrated (2). According to the current evidence, SARS-Cov-2 commonly involves individuals aged 30-80 years and has low mortality in healthy individuals but can be life-threatening, resulting in severe illness and even death due to sepsis, acute respiratory distress syndrome (ARDS) and multi-organ failure (2).

Pemphigus vulgaris is a potentially life-threatening autoimmune bullous disease affecting the skin and mucosa and is caused by autoantibodies directed against desmoglein 1 and desmoglein 3 adhesion molecules of the epidermis (3, 4). Severe cases of PV represent a true medical emergency (5). Since the public announcement of the COVID-19 outbreak, several concerns have been raised by dermatologists as well as pemphigus patients who take immunosuppressive drugs. These concerns include the need for proper disease control with minimal immune suppression to avoid possible fatal outcomes. It is also crucial to understand how the underlying mechanisms in COVID-19 (e.g. cytokine release storm leading to interstitial pulmonary inflammation, extensive lung damage and acute respiratory distress syndrome) (6) could affect those auto-immune diseases such as pemphigus.

In this paper, we review the literature on the common treatments of pemphigus with a focus on lessons from similar epidemics to find a proper suggestion to manage pemphigus in the COVID-19 pandemic era.

**Systemic corticosteroids**

Historically, systemic corticosteroids, usually oral prednisone alone or in combination with immunosuppressive drugs, have been used as the mainstay treatment in pemphigus vulgaris (7). Although these agents have led to substantial improvement in the prognosis of the disease, treatment complications, especially the risk of infections, remain major areas of concern (8, 9). When used as pulse therapy, steroids may lead to cardiac side effects (10, 11). This concern becomes even more pronounced during the epidemic of some infectious agents, including the coronavirus.

Considering the effect of systemic corticosteroids on suppressing inflammation and the presence of lung inflammation induced by host immune responses in influenza, SARS-CoV, MERS-CoV, and SARS-CoV-2 infections, these therapeutic agents have been of interest to physicians during the outbreaks of these infections (2, 12). Existing clinical data have not confirmed the beneficial effect of corticosteroids in treatment of respiratory infections due to SARS-CoV, or MERS-CoV (12). The observational studies had reported increased mortality and secondary infection rates in influenza, impaired clearance of SARS-CoV and MERS-CoV, and complications of corticosteroid therapy (e.g. diabetes, avascular necrosis, and steroid-induced psychosis) in survivors (13, 14). Therefore, not only does the role of steroids in the treatment of acute lung injury in these viral infections remain controversial, but also this treatment may be harmful in patients with 2019-nCoV infection (12, 15).

Currently, pandemic-related emotional stress, decreasing the dose of immunosuppressive medications for fear of COVID-19 and eventually getting this infection may be considered as exacerbating factors or triggers for pemphigus vulgaris (16). Therefore, strict adherence to health principles and avoiding emotional stress while continuing the treatment protocol recommended by dermatologists may help prevent exacerbation or recurrence of pemphigus. 

**Rituximab**

Rituximab (RTX) is a chimeric monoclonal anti-CD20 antibody that causes depletion of CD20-expressing B cells (17, 18). Early treatment with rituximab has resulted in higher remission rates, long term clinical response, lower incidence of serious adverse events and rapid prednisone tapering compared to old immunosuppressive therapies making its approval as a first-line therapy in pemphigus possible (19). Rituximab is generally considered safe in patients with pemphigus vulgaris and serious infections, while reported, are rare. Although single RTX infusions do not seem to impair memory responses against known pathogens (20), patients may exert a defective immune reaction against new pathogens and life-threatening infections, including sepsis, have been reported following RTX treatment (21). Opportunistic infections such as cytomegalovirus infection and Pneumocystis pneumonia (PCP), although extremely rare and limited to sporadic case reports, have been reported (22, 23). The risk of reactivation of hepatitis B and C viruses as well as tuberculosis has also been reported (17). 

It should be noted that protective humoral immunity in the central nervous system (CNS) requires peripheral CD19-dependent germinal center formation following neurotrophic coronavirus infection (24) and there are evidence that show SARS-CoV-2 targets CNS (25). Although the expression of ACE 2 (the host cell-surface receptor for SARS-CoV-2 envelope spike glycoprotein) in the brain has been proposed, the exact mechanism of CNS involvement in COVID-19 is not known, yet (25). Accordingly, since the characteristic of the immune response against SARS-CoV-2 has not been identified, the potential risks of treating pemphigus with rituximab should be taken into account. Special attention must be paid to close monitoring of the patients and tapering concurrent corticosteroids to minimize susceptibility to life-threatening infections. This risk should be weighed against the prominent and effective role of RTX in treatment of pemphigus and reducing the number of follow up visits (19).

**Other immunosuppressive agents**

 Most guidelines suggest azathioprine (AZA) or mycophenolate mofetil (MMF) as first-line steroid-sparing agents for treatment of pemphigus rather than other adjuvant immunosuppressants (18). Nevertheless, different variables such as age and comorbidities of the patients, dermatologist’s personal experience and costs need to be considered since other drugs, such as cyclophosphamide, also show efficacy (8). It should be noted that in comparison to corticosteroid alone, these drugs mainly exert a corticosteroid-sparing effect that leads to a reduction in the risk of relapse by 29% rather than achieving remission (26). Currently, data regarding the administration of these drugs during COVD-19 are scarce and inconclusive. We recommend that these drugs only be discontinued in proven cases of COVID-19. It is noteworthy that there are reports of atypical presentation of Middle East respiratory syndrome virus (MERS-CoV) in patients who received immunosuppressive drugs; therefore, careful monitoring of patients for atypical symptoms should be taken into account (27).

The administration of intravenous immunoglobulin (IVIg) is a therapeutic option in patients with severe/refractory PV (28). Since IVIg might be the safest immunomodulator for long-term use in all age groups (29), treatment with IVIg has been proposed as a potential option for COVID-19 (6) but data are scarce and controversial. In pemphigus patients with COVID-19 and flare of PV, this therapeutic option can be considered but should be weighed against possible side effects such s thromboembolism in severely ill patients (30).

**Recommendations**

**Figure1:**
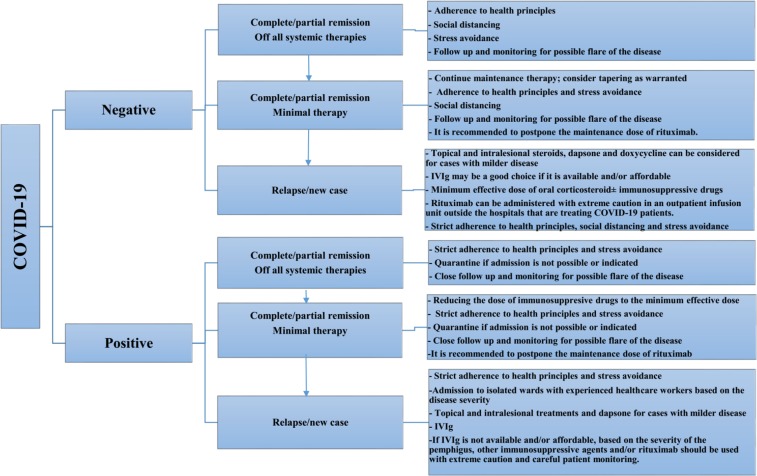
The proposed algorithm for management of pemphigus patients during the outbreak of COVID-19. Off all systemic therapies: the patient has not been taking any systemic therapy for at least 2 months. Minimal therapy: the patient has been on maintenance therapy [prednisolone (or the equivalent) ≤ 10mg/d and/or minimal adjuvant therapy and/or topical corticosteroids for at least 2 months). Partial remission: There are transient lesions that heal within a week without additional treatment. Complete remission: There is no stable or new lesion. Relapse: The extension of stable lesion(s) and/or the development of ≥ 3 new lesions per month that do not disappear within 1 week without additional treatment IVIG: intravenous immunoglobulin(18)

Patients with pemphigus need proper treatment for their underlying condition; however, during the outbreak of COVID-19, these patients may carry the risk of severe infections with disastrous outcomes. Figure 1 is a proposed algorithm for management of patients with pemphigus during the COVID-19 outbreak based on the former consensus on treatment of pemphigus and the current evidence on COVID-19.

 The following issues should be particularly considered.

- Clear information should be given to patients and caregivers to avoid panic and mismanagement.
- Application of teledermatology resources for close follow up and monitoring of the patients to minimize patient’s referral to healthcare centers could be very helpful (31).
- Patients should be followed at regular intervals with a focus on screening the patients for symptoms of COVID-19.
- Healthcare professionals who are in close contact with these patients should be monitored and be vigilant in using recommended precautions and proper ethical teledermatology practice (31).
- Psychological tolerance of patients during quarantine, especially in those who receive high doses of corticosteroids leading to mood swings, should be monitored and possible coping strategies should be discussed.
- The possible flare of the underlying condition should be seriously considered in cases of drug discontinuation without reasonable indication.
- Patients with milder disease can be treated less aggressively with local steroids (topical or intralesional), dapsone and doxycycline (7).
- If indicated, it is better to administer RTX in an infusion center outside the hospitals that are treating COVID-19 patients.
- With regard to the current and emerging treatments of COVID-19 such as anti-malaria drugs (32) and IL6 inhibitors (33), dermatologists have to consider possible drug interactions or more rigorous prophylactic strategies in PV patients, respectively.

## Conclusion:

We are currently in the midst of a pandemic and evaluating the effect of COVID-19 on the population of susceptible patients suffering from auto-immune diseases like pemphigus is essential. Many patients with PV may present to emergency rooms needing emergent care. 

The effect of many of the drugs used for treatment of Pemphigus vulgaris (PV) on COVID-19 is not clear. We also do not have data on the impact of this autoimmune disease, which may involve the mucous membranes, on the acquisition or course of COVID-19. Despite these limitations, the evidence on best ways to manage patients with underlying conditions, such as pemphigus, during the outbreak of COVID-19 is evolving and the data is updated every day. We hope the issues brought up by this paper can help physicians to make the best decisions for their patients. 
